# HIV testing week 2015: lowering barriers for HIV testing among high-risk groups in Amsterdam

**DOI:** 10.1186/s12879-017-2617-0

**Published:** 2017-08-01

**Authors:** M. Bartelsman, I. K. Joore, J. E. van Bergen, A. A. Hogewoning, F. R. Zuure, M. G. van Veen, On behalf of the HIV Transmission Elimination AMsterdam (H-TEAM) initiative, J. E. A. M. van Bergen, F. Deug, M. Heidenrijk, J. M. Prins, M. Prins, P. J. Smit, M. van der Valk, P. Reiss, G. J. de Bree, E. Hoornenborg, U. Davidovich, S. E. Geerlings, A. Oomen, A. van Sighem, W. Zuilhof, R. C. A. Achterbergh, M. van Agtmael, J. Ananworanich, D. Van de Beek, G. E. L. van den Berk, B. Berkhout, B. Berretty, D. Bezemer, W. L. Blok, M. Bomers, C. A. B. Boucher, W. Brokking, D. Burger, K. Brinkman, M. de Bruin, E. L. M. Op de Coul, B. O’Dell, L. Dellemann, C. Ester, Y. T. van Duijnhoven, M. Dijkstra, A. van Eeden, S. van Elsen, L. Elsenburg, P. H. J. Frissen, T. B. H. Geijtenbeek, M. H. Godfried, A. Goorhuis, M. Groot, R. A. Gruters, C. A. Hankins, A. Heijne, J. J. van der Helm, M. Hillebregt, A. Hogewoning, J. W. Hovius, I. K. Joore, N. A. Kootstra, R. A. Koup, F. P. Kroon, F. Lauw, K. Lettinga, T. Mahmoudi, J. T. van der Meer, S. van Meeteren, J. Mulder, F. J. Nellen, H. Nobel, A. O. Pasternak, E. Peters, T. van der Poll, M. F. Schim van der Loeff, W. E. M. Schoute, C. Rokx, M. S. van Rooijen, G. J. Sonder, M. G. van Veen, J. Veenstra, A. Verbon, F. Verdult, D. Verhagen, G. Visser, H. J. de Vries, J. de Vocht, S. Vrouenraets, M. van Vugt, W. J. Wiersinga, F. W. Wit, L. R. Woittiez, S. Zaheri, M. C. van Zelm, F. R. Zuure

**Affiliations:** 1 STI Outpatient Clinic, Public Health Service of Amsterdam (GGD Amsterdam), Amsterdam, the Netherlands; 2 Department of General Practice/Family Medicine, Division of Clinical Methods and Public Health, Academic Medical Center, Amsterdam, the Netherlands; 3 STI AIDS Netherlands (Soa Aids Nederland), Amsterdam, the Netherlands; 4 Epidemiology & Surveillance Unit, Centre for Infectious Disease Control, National Institute of Public Health and the Environment (RIVM), Bilthoven, the Netherlands; 5 Department of Infectious Diseases Research and Prevention, Public Health Service of Amsterdam (GGD Amsterdam), Amsterdam, the Netherlands; 6 Department of Internal Medicine, Center of Infectious diseases and Immunology Amsterdam (CINIMA), Academic Medical Center, Amsterdam, the Netherlands; 7 Public Health Service of Amsterdam, Department of Infectious Diseases, STI Outpatient Clinic, Weesperplein 1, 1018 WZ Amsterdam, the Netherlands

**Keywords:** HIV, HIV testing week, Point-of-care test, Rapid test, Msm, Migrants

## Abstract

**Background:**

Evaluation of the HIV Testing Week (HTW) 2015 in Amsterdam: the number of (positive) tested persons, characteristics and testing history of the tested population, the differences in attendance per location and the healthcare workers’ experiences and opinions concerning the HTW.

**Methods:**

The HTW took place from 28 November till 4 December 2015. Anonymous HIV rapid testing (INSTI™ HIV1/HIV2 Ab test or Determine™ HIV-1/2 Ag/Ab test) was offered free of charge at four hospitals, 12 general practitioner (GP) clinics, a sexually transmitted infections (STI) clinic, a laboratory, sites of a community-based organisation, and at outreach locations. Home-based testing (OraQuick® In-Home HIV Test) was offered online. The focus was to motivate two groups to test: men who have sex with men (MSM) and non-Western migrants. Questionnaires regarding participant’s characteristics and HIV testing history were collected. Also healthcare workers were asked to complete a questionnaire evaluating the HTW.

**Results:**

In total, 1231 participants were tested. With three positive HIV tests, the detection rate was 0.3% (95%CI 0.26–0.37). Of all participants, 24.7% (304/1231) were MSM. Respectively, 22.3% (275/1231) and 15.7% (193/1231) were first- and second-generation migrants from a non-Western country. Altogether, 56.7% (698/1231) of participants belonged to one of the targeted risk groups. For 32.7% (402/1231) of participants, it was the first time they received testing, and 35.1% (432/1231) were tested more than 1 year ago. Among MSM 13.2% were tested for the first time, among first- and second-generation non-Western migrants this percentage was significantly higher at 27.2% and 33.5% respectively (*p* < 0.01). The number of tested participants per location varied widely, especially between GP clinics (range 3–63). Healthcare workers were positive about the HTW: about half (46.2%) stated they would more readily offer an HIV test following their experience with the HTW.

**Conclusions:**

This was the first time the Amsterdam HTW was organised on such a large scale. The majority of the tested population belonged to one of the targeted risk groups and received testing either for the first time or for the first time in over a year. It is important to further build upon the experiences of the HTW and offer free of charge low-threshold HIV testing more structurally. An evaluation of cost-effectiveness is also warranted for future editions of the HTW.

## Background

In the Netherlands an estimated 22,100 individuals are living with HIV, of whom about 2700 (12%) are unaware of their infection [1]. A study has demonstrated the estimated percentage that is unaware among migrant groups is even higher: 33% and 25% among HIV-infected migrants from sub-Saharan Africa and the Caribbean respectively [2]. Moreover, 44% of those testing positive enter care late [1].

Detecting and treating HIV-infected individuals at an early stage is an important public health challenge: besides the prognostic benefits for the individual, there is growing evidence that early detection and treatment is crucial for the reduction of HIV transmission among the population [3–5].

HIV testing is offered in different settings in the Netherlands. At general practitioners (GP) clinics and in hospitals HIV testing is offered on indication or request only. Although costs of diagnostics are covered by health insurance, people with no other healthcare expenses have to pay the tests themselves because these costs are set off against the obligatory deductible excess. Public sexually transmitted infections (STI) clinics provide free-of-charge testing for sexual transmitted infections and HIV but only serve high-risk groups, like men who have sex with men (MSM) and non-Western migrants. Community-based free-of-charge HIV testing is offered in Amsterdam by a non-governmental organization. In addition, there are commercial providers who offer HIV home-collection tests, and in 2014–2016 HIV self-tests were sold in the Netherlands in a pilot programme (www.time2test.nl). Since 2004 all pregnant women are offered screening for HIV in prenatal care clinics.

Most national and international guidelines advocate for a more proactive HIV testing policy for high-risk groups [6–9]. Yet, there are barriers for its implementation. A recent study conducted in primary healthcare settings in the Netherlands revealed that no HIV test was performed in one-third of STI related consultations with high-risk groups [10].

Since 2015, multiple stakeholders engaged in HIV prevention and treatment joined forces on the ‘HIV Transmission Elimination Amsterdam’ (H-TEAM) initiative (www.hteam.nl), with the aim of reducing the HIV epidemic in Amsterdam and improving the prognosis for people living with HIV [11]. One of the H-TEAM’s initiatives was an HIV Testing Week (HTW) in 2015 in Amsterdam. Anonymous HIV rapid testing was offered free of charge at various clinical and non-clinical healthcare locations throughout the city of Amsterdam.

The goals of the HTW were to create awareness by emphasising the importance of early testing among both professionals and inhabitants of Amsterdam, and to normalise and increase proactive HIV testing and the detection of new HIV infections. The HTW campaign primarily targeted two main risk-groups: MSM and non-Western migrants.

The primary aim of this study was to evaluate whether the HTW was an effective strategy to increase testing and detect new infections among the targeted risk groups. In addition, experiences with the HTW were evaluated among the participating healthcare workers.

## Methods

### Test locations

The HTW took place in the week of World AIDS Day, from 28 November till 4 December 2015.

A total of four hospitals, 12 general practitioner (GP) clinics, the sexually transmitted infections (STI) outpatient clinic of the Public Health Service (PHS) of Amsterdam, a primary healthcare laboratory (SHO), and a community-based organisation (AIDS Health Care Foundation (AHCF)) opened their doors to anyone who wanted to receive testing.

In addition, outreach testing was organised at different locations. Most of the outreach activities were in the south-eastern quarter of Amsterdam, an area with a high proportion of non-Western migrants originating from HIV-endemic countries (mostly Caribbean and African countries). An extra testing site was created at the south-eastern district’s office and a special mobile testing bus visited various open-air markets. A home-based testing pilot programme (project website www.time2test.nl) also participated and offered free of charge HIV self-tests during the HTW. This programme aimed to develop and evaluate an online service that provided a reliable HIV self-test using oral fluid People could order the kit via the website or, using a pick-up code, at different pick-up locations in the Netherlands. Pre- and post-test counseling was provided online, and a telephone number was available for personal assistance if needed.

### HIV rapid tests and testing procedure

The majority of the organisations (GP clinics, STI outpatient clinic, SHO, AHCF, AMC, and the Slotervaart hospital) used the INSTI™ HIV1/HIV2 Rapid Antibody test (Biolytical TM, Laboratories Inc., Richmond, BC, Canada). The INSTI test is an HIV antibody test that yields results within 60 s by obtaining blood with a finger prick. The other two hospitals (OLVG and DC clinic) preferred use of the Determine™ HIV-1/2 Ag/Ab Combo test (Alere Inc., San Diego, USA) because of their routine use of this test. The home-based self-test was an oral HIV rapid test (OraQuick® In-Home HIV Test, OraSure Technologies, Inc., Bethlehem, USA) and could be ordered online and picked up at the PHS of Amsterdam as part of the Time2test project.

In case of a positive rapid test confirmation, testing was done at a central laboratory (of the PHS or one of the hospitals) and when confirmed positive, a participant was directly linked to a centre with specialised HIV care. In Time2test project, persons who tested positive could download a referral letter to regular health care facilities for confirmation testing from the project website. No data were collected from health care professionals regarding follow-up in care for these participants.

### Training of staff

In the weeks preceding the HTW, training was organised for all healthcare workers of the participating locations. During the training, healthcare workers practiced using the INSTI-test, conducting pre- and post-test counselling, and were briefed about the testing protocol and questionnaires that should be offered to all participants.

### Campaign

In cooperation with the Dutch AIDS Foundation (AIDS Fonds/SOA AIDS Nederland) a multi-media campaign was launched in the weeks before the HTW. A website (www.HIVtestweek.nl) was created with general information about the HTW and contact information for all the different testing locations. Participants could search a map for nearby testing locations using their postal code. Posters advertising the HTW website were placed in 100 bus shelters throughout Amsterdam. The central aim of the campaign was to normalise and lower the stigma of HIV testing.

All 497 Amsterdam GP’s received an information package including materials and the latest scientific information. A total of 25,000 flyers and 4000 posters were distributed among 205 GP clinics, 4 hospitals, and 3 pharmacies. Also, the outreach teams of the AHCF and the STI outpatient clinic of Amsterdam distributed about 1000 flyers and 50 posters in gay venues in the centre of Amsterdam and in shops, markets, community centres, and churches in the multicultural south-eastern quarter of Amsterdam.

Online marketing and advertising were mainly targeted at the two risk groups by placing banners and advertorials on specific websites for MSM and ethnic minorities. Traditional media also paid attention to the HTW: articles appeared in newspapers and several items were broadcast on local and national radio and television. Before the launch of the campaign a community advisory group was formed. The group consisted of 12 representatives of African and Caribbean communities who provided feedback on the campaign and organisation of the HTW.

### Evaluation HTW 2015

We evaluated the number and the characteristics of the tested participants, the detection rate and the HTW experience of the participating healthcareworkers. In addition, we assessed the number of participants per testing location in relation to promotional activities organized by these locations.

### Questionnaires participants

For evaluation purposes, all participants were asked to complete a questionnaire regarding their demographic background, sexual preference, HIV testing history, and more general questions about the HTW campaign. The questionnaires were available in English and Dutch.

In case of a positive HIV rapid test, the participant was asked to sign an informed consent form giving researchers of the PHS of Amsterdam permission to collect the confirmation test results. Participants who opted for the home-based test were asked to complete the questionnaire when they picked up the test at the PHS.

### Questionnaires healthcare workers

After the HTW, an evaluation questionnaire was sent by mail to all testing locations (with the exception of the PHS and the AHCF where HIV rapid testing is practised all year round). The aim of this questionnaire was to evaluate the opinion of the healthcare workers regarding the HTW’s organisation and campaign, their experience with the newly introduced rapid HIV test (the latter was not evaluated at two hospitals where another HIV rapid test was used), and whether or not they had initiated extra promotional activities. At every location one healthcare worker who was the most involved during the HTW was requested to complete the questionnaire.

### Data analysis

Descriptive statistics were used to analyse baseline characteristics. Differences in testing history between risk groups were compared using the Pearson’s chi-square test. We considered a *p*-value <0.05 as statistically significant. Analyses were performed with SPSS package version 21 (SPSS Inc., Chicago, Illinois, USA).

The sexual preference of men was based on self-reported sexual behaviour in the questionnaires.

The definition of Western and non-Western migrants was based on the definition used by the Dutch central agency for statistics (CBS) [12]. Also the distinction between a first-generation migrant (person is born in a country other than the Netherlands) and a second-generation migrant (person is born in the Netherlands but at least one of the parents is born in a country other than the Netherlands) was based on the definition of the CBS.

A country was considered HIV-endemic when the estimated HIV prevalence was higher or equal to 1.0 (based on data of the United Nations Programs on HIV/AIDS (UNAIDS) [13].

## Results

### Number of tested participants and positive rapid tests

During the HTW a total of 1231 participants were tested: 24.6% (303/1231) at a hospital, 22.8% (281/1231) at the STI outpatient clinic, 22.3% (275/1231) at the AHCF and the outreach sites, 21.9% (270/1231) at the GP clinic, and 8.1% (100/1231) through the home-based testing project ‘Time2test’. As shown in Table 1 the number of tested participants per location varied widely, especially between GP clinics (range 3–63 participants).

**Table 1:** Number of participants and HIV positive test per test location, HIV Testing Week 2015, Amsterdam, the Netherlands

Type of location	Test locations	Number of participants (% of subtotal)	Number of HIV positive tests (% of total)
GP clinics	A	3 (1.1%)	0
	B	6 (2.2%)	0
	C	36 (13.3%)	0
	D	30 (11.1%)	0
	E	8 (3.0%)	0
	F	3 (1.1%)	0
	G	63 (23.3%)	0
	H	11 (4.1%)	0
	I	54 (20.0%)	0
	J	10 (3.7%)	0
	K	18 (6.7%)	0
	L	28 (10.4%)	1/28 (3.6%)
	Subtotal GP clinics	270 (21.9%)	1/270 (0.4%)
Hospitals	AMC	42 (13.9%)	0
	Slotervaart hospital	35 (11.6%)	0
	OLVG	206 (68.0%)	1/206 (0.5%)
	DC Clinic	20 (6.6%)	0
	Subtotal hospitals	303 (24.6%)	1/303 (0.3%)
Outreach	PHS outreach	28 (10.2%)	0
	Aids Healthcare Foundation	247 (89.8%)	0
	Subtotal outreach	275 (22.3%)	0
Municipal Health Service	PHS STI outpatient clinic	281 (22.8%)	1/281 (0.4%)
Laboratory	SHO	2 (0.2%)	0
Home-based test	Time2test	100 (8.1%)	Unknown
Total		1231 (100%)	3/1131^a^ (0.3%)

^a^of 100 participants who did the home-based test (Time2test) the test results were unknown

With three positive HIV test results through rapid testing, the overall detection rate was 0.3% (3/1131, 95%CI 0.26–0.37). Two participants gave permission for collection of their confirmation test result: both were confirmed positive for HIV type 1 and referred directly to an HIV specialist for further care. The third participant who was tested positive did not give permission for collection of the confirmation test results; this participant tested positive with a rapid test at the hospital where blood for confirmation tests was taken directly.

For 10.7% (132/231) of the participants the test result was unknown, of these, 100 were home-based test participants and 32 were participants for whom the test result was not recorded by the healthcare worker. The latter group was included in the denominator of the calculations for the detection rate assuming these results were negative.

### General characteristics of participants

The median age of all the participants was 33 years (IQR 26–45 years; range 16–81 years) (see Table 2). The gender distribution was as follows: 58.2% (716/1231) male, 39.3% (484/1231) female, 0.2% (3/1231) transgender, and 2.3% unknown (28/1231). Of all participants, 24.7% (304/1231) were MSM, including 13.3% homosexual men (164/1231) and 11.4% bisexual men (140/1231).

**Table 2:** General characteristics of the participants of the HIV Testing Week 2015, Amsterdam, The Netherlands

General characteristics (*N* = 1231)	Years (IQR^a^) n/N (%)
Median age	33.0 (26–45)
Gender	
Male	716 (58.2%)
Female	484 (39.3%)
Transgender	3 (0.2%)
Unknown	28 (2.3%)
Sexual orientation men^b^	
Men who have sex with men:	304 (24.7%)
Homosexual men	164 (13.3%)
Bisexual men	140 (11.4%)
Heterosexual men	400 (32.5%)
Unknown	12 (1.0%)
Ethnicity	
Dutch native	497 (40.4%)
First-generation^c^migrant from a non-Western^d^ country	275 (22.3%)
Second-generation^e^ migrant from a non-Western country	193 (15.7%)
First-generation migrant from a Western country	101 (8.2%)
Second-generation migrant from a Western country	26 (2.1%)
Unknown	139 (11.3%)

^a^interquartile range

^b^% of total study population

^c^first-generation migrant = participant is born in a country other than the Netherlands

^d^according to a country list of the Dutch central agency of statistics (CBS)

^e^second-generation migrant = at least one of the parents of the paricipant is born in a country other than the Netherlands

In total, 40.4% (497/1231) of the participants were native Dutch, 22.3% (275/1231) were first-generation migrants from a non-Western country, 8.2% (101/1231) were first-generation migrants from a Western country, 15.7% (193/1231) were second-generation migrants from a non-Western country, 2.1% (26/1231) were second-generation migrants from a Western country, and of 11.3% (139/1231) data were lacking concerning country of origin. Of the non-Western migrants, 60.5% (283/468) originated from an HIV endemic country.

Altogether, 56.7% (698/1231) of the participants belonged to one of the HTW targeted risk groups (MSM and/or first- or second-generation migrants from a non-Western country).

### Testing history

For 32.7% (402/1231) of the participants, it was the first time they were tested for HIV, 25.2% (310/1231) had been tested less than a year ago, 35.1% (432/1231) were tested more than 1 year ago, 1.4% (17/1231) had been previously tested but information about the moment of testing was unknown, and of the remaining 5.7% (70/1231) it was unknown whether they had ever been tested.

Testing history differed by risk group: 13.2% (40/304) of the MSM had never been tested for HIV, of the first- and second-generation non-Western migrants (and not MSM) a significantly higher percentage of 27.2% (61/224) and 33.5% (57/170), respectively, had never been tested for HIV (*p* < 0.01).

### Opinions and experiences of participating healthcare workers

In total, healthcare workers of 14 of the 17 test locations that were sent an evaluation questionnaire (12 GP clinics, four hospitals, and one primary healthcare laboratory) responded.

Overall the healthcare workers that completed the questionnaire were positive about the organisation and campaign of the HTW; an average rating of 8 was given (on a scale of 0 to 10). A majority (85.7%) were willing (sure to most likely) to participate in the next HTW.

The healthcare workers of the locations that used the INSTI test (*n* = 12) were positive about the ease of use of the test (an average rating of 7.8) and about the whole procedure related to a direct result (an average rating of 8.6). A majority (84.6%) of the health care workers was positive about the idea of using the INSTI test more regularly in practice. Almost half of the healthcare workers (46.2%) would more readily offer their patients an HIV test (sure to most likely) after their experience with the HTW. The extent to which healthcare workers of the locations had initiated activities to attract more participants varied between the locations. The three GP clinics with the highest attendance (36 to 51 participants) had posted information on their websites and/or had invited their patients by email., whereas the GP clinics with a lower attendance (3–30 participants) did not report extra promotional activitites.

## Discussion

This was the first time the Amsterdam HTW was organised on such a large scale. Although the number of tested persons varied widely among the different test locations, we reached a total of 1231 persons. More than half (56.7%) of the tested population belonged to one of the targeted risk groups. A substantial new group of people was reached for HIV testing by the HTW: about one-third of the tested participants received HIV testing for the first time and about one-third for the first time in over a year. Three persons were newly diagnosed with HIV through rapid testing (0.3%), who all tested for HIV for the first time. In general, the participating healthcare workers were positive about the HTW. The majority of healthcare workers that used the rapid test indicated to be willing to use this HIV test more regularly in practice and about half of this group would offer an HIV test more readily in the future.

Although the absolute number of three positive HIV test results is not high, the detection rate of 0.3% was as high as the annual detection rate among high-risk visitors of the STI outpatient clinic in Amsterdam [14]. It is comparable with testing weeks in other countries. In England, a national HTW was organised at various outpatient and emergency departments in 2014 and a detection rate of 0.12% was found [15]. In the USA, a National HIV Testing Day (NHTD) is organised every year and in 2010 a detection rate of 0.7% was reported in the week around the NHTD [16]. Yet, measuring the success of the HTW by comparing the detection rates between countries is not that simple: in England and the Netherlands the detection rates were based on very small numbers of detected infections (in England there were also three newly diagnosed). Moreover, the adult background prevalence of HIV in the USA (0.4%–0.9%) is higher than in England and the Netherlands (0.2%) [17].

One of the aims of the HTW was to increase awareness concerning HIV testing among professionals. It is not clear whether the HTW influenced healthcare workers’ HIV testing behaviour, but they did provide positive feedback regarding the intention to perform more proactive HIV testing in their daily routine.

A recent analysis of the Centers for Disease Control and Prevention (CDC) revealed a significant increase in HIV testing and new HIV diagnoses around the NHTD in the USA [18]. For future HTW’s in Amsterdam we also plan to evaluate whether the HTW will have an enduring effect on provider-initiated testing. In addition, the impact on awareness concerning HIV testing among the population and the cost-effectiveness of the HTW need further investigation.

Community involvement of different cultural ethnic organisations played an important role in the organisation and campaign of this HTW. As shown in the results, about one-third of the tested non-Western migrants had never been tested. Available research suggests that migrants originating from non-Western countries have a higher vulnerability for HIV and are not using HIV testing facilities optimally, namely testing late [19]. Among migrants there are more barriers to HIV testing, such as a lower risk perception, lack of general knowledge about HIV, fear of the consequences of a positive HIV result, negative social norms, and costs of HIV tests [20–25]. Moreover, structural preconditions like the availability of affordable low-threshold testing in neighbourhoods with a high concentration of migrants are essential [19]. There is still much to improve concerning low-threshold testing in these kinds of neighbourhoods in Amsterdam, like the multicultural south-eastern district. Besides provider-initiated testing at GP clinics and the STI outpatient clinic, community-based testing needs more attention. The HTW was an impulse to enhance pro-active HIV testing among migrants. After the HTW the STI outpatient clinic intensified STI testing among migrants by expanding the STI/sexual health care in a satellite clinic in the south-eastern district of Amsterdam.

The HTW not only has to be seen as an intervention in itself, but also as a catalyst for new strategies which could accelerate HIV testing, create awareness, and lower barriers for testing among specific risk groups and professionals. A new strategy to lower barriers for testing could include offering HIV self-tests online or at pharmacies. An evaluation of a recent trial using self-tests (Time2test) that was offered during the HTW demonstrated it was successful in reaching first-time and infrequent testers [26]. Other studies investigating home-based and in-pharmacy testing in different European countries and the USA also show promising results [27–30]. In countries like France, the USA, and the United Kingdom reliable self-tests are now approved to be sold structurally at pharmacies or online [31, 32]. Unfortunately the Dutch government still does not allow professional parties to offer reliable HIV self-tests online.

## Conclusions

In conclusion, the HTW was an important additional step in creating awareness and lowering the barriers for proactive HIV testing. The results showed that the majority of the tested population belonged to one of the targeted risk groups and that a new group of people was reached that had never been tested. Now it is time to further build upon the experiences of the HTW and have free of charge low-threshold HIV testing offered more structurally by healthcare professionals and in community-based settings.

## Abbreviations

AHCF: AIDS health care foundation
CDC: Centers for disease control and prevention
GP: General practitioner
H-TEAM: HIV transmission elimination amsterdam
HTW: HIV testing week
MSM: Men who have sex with men
NHTD: National HIV testing day
PHS: Public health service
STI: Sexually transmitted infections
